# Supplementary material for: Asynchronous firing and off states in working memory maintenance
Source: Cell Rep. Author manuscript; Available in PMC 2026 Feb 28. (PMC12949571; doi:10.1016/j.celrep.2025.116764)
Supplement: 1 [file NIHMS2142937-supplement-1.pdf]

Cell Reports, Volume *45*

Supplemental information

Asynchronous firing and off states

in working memory maintenance

Rana Mozumder, Zhengyang Wang, Wenhao Dang, Junda Zhu, Benjamin M. Hammond, Anna Machado, and Christos Constantinidis

SUPPLEMENTAL INFORMATION

Document S1. Figures S1–S10

SUPPLEMENTAL FIGURES

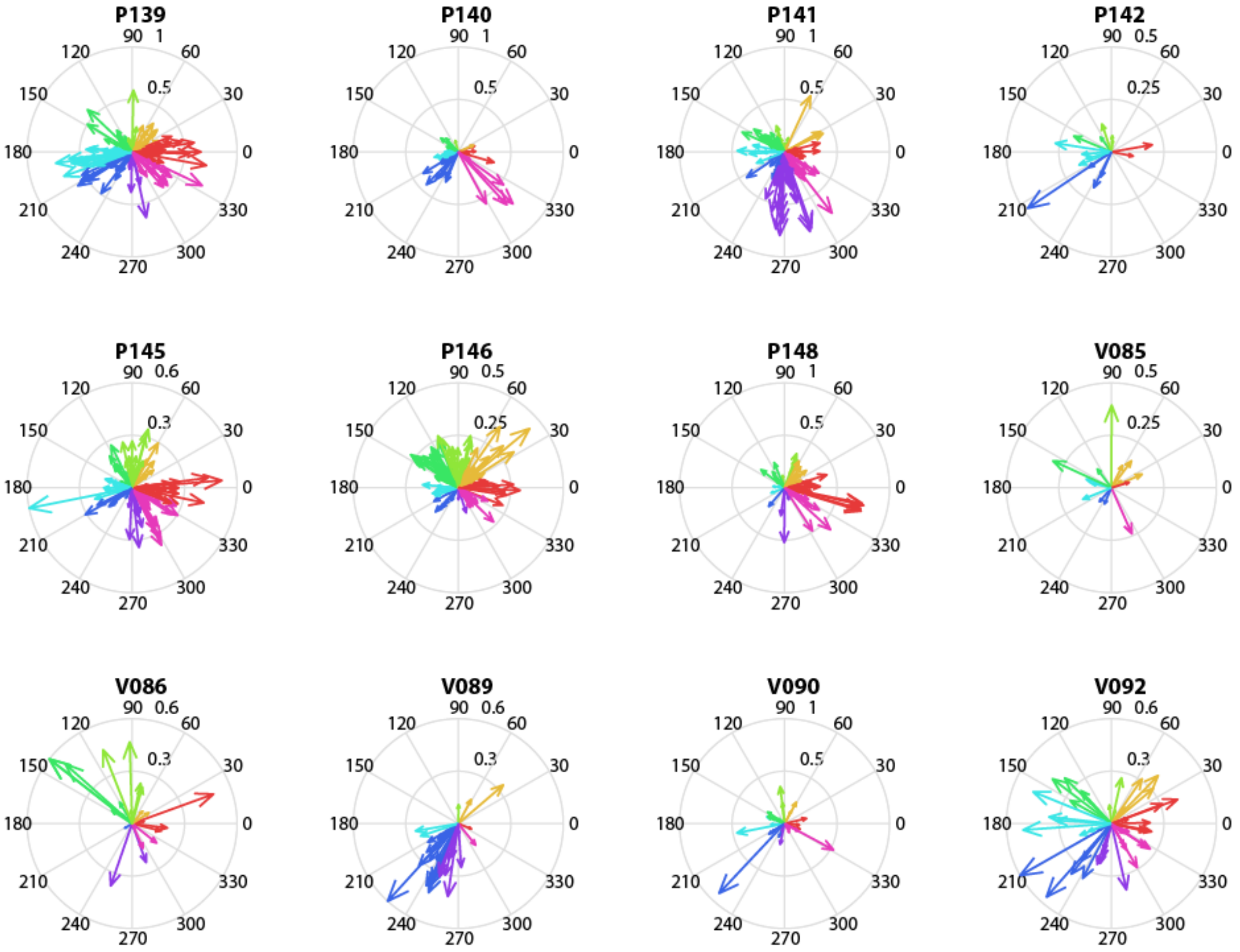

Figure S1. Population compass plots, related to Figure 1. Plots illustrate the tuning of all delay-selective neurons across all recording sessions analyzed (in addition to session P138, shown in Fig. 1).

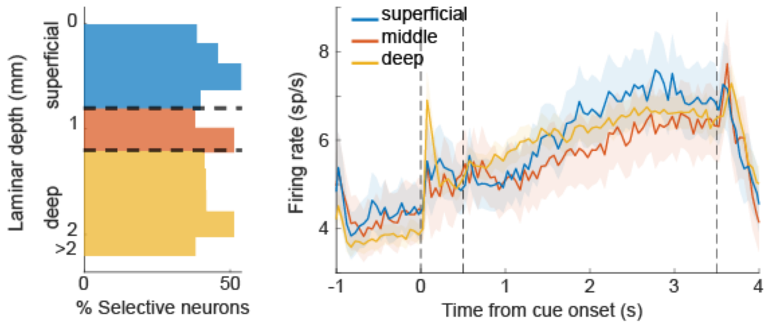

Figure S2. Laminar distribution of delay activity, related to Figure 1. (A). Histogram represents the percentage of selective neurons recorded at each depth relative to the top of the cortex (depth 0) that exhibited tuned, persistent activity. B. Population PSTH for neurons divided in superficial (0-800 µm, n=149), middle (800-1200 µm, n=119) and deep layers (>1200 µm from the top of the cortex, n=667).

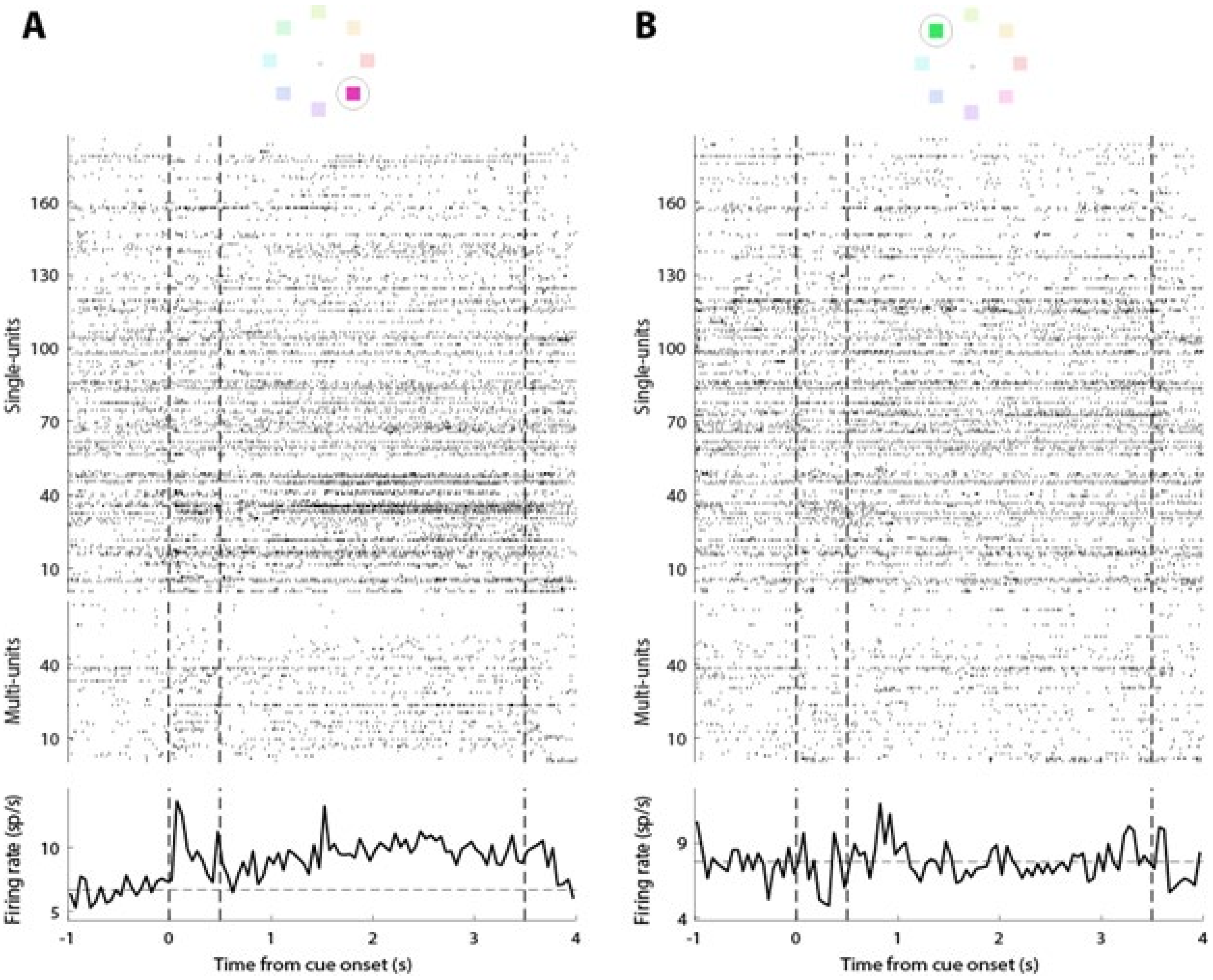

Figure S3. Examples of persistent and non-persistent delay activity, related to Figure 1. Raster plot and PSTH for two example trials (left: 315º cue stimulus, right: 135º cue stimulus) for single and multi-units recorded simultaneously from a recording session (P138). The conventions are the same as in Figure 1G.

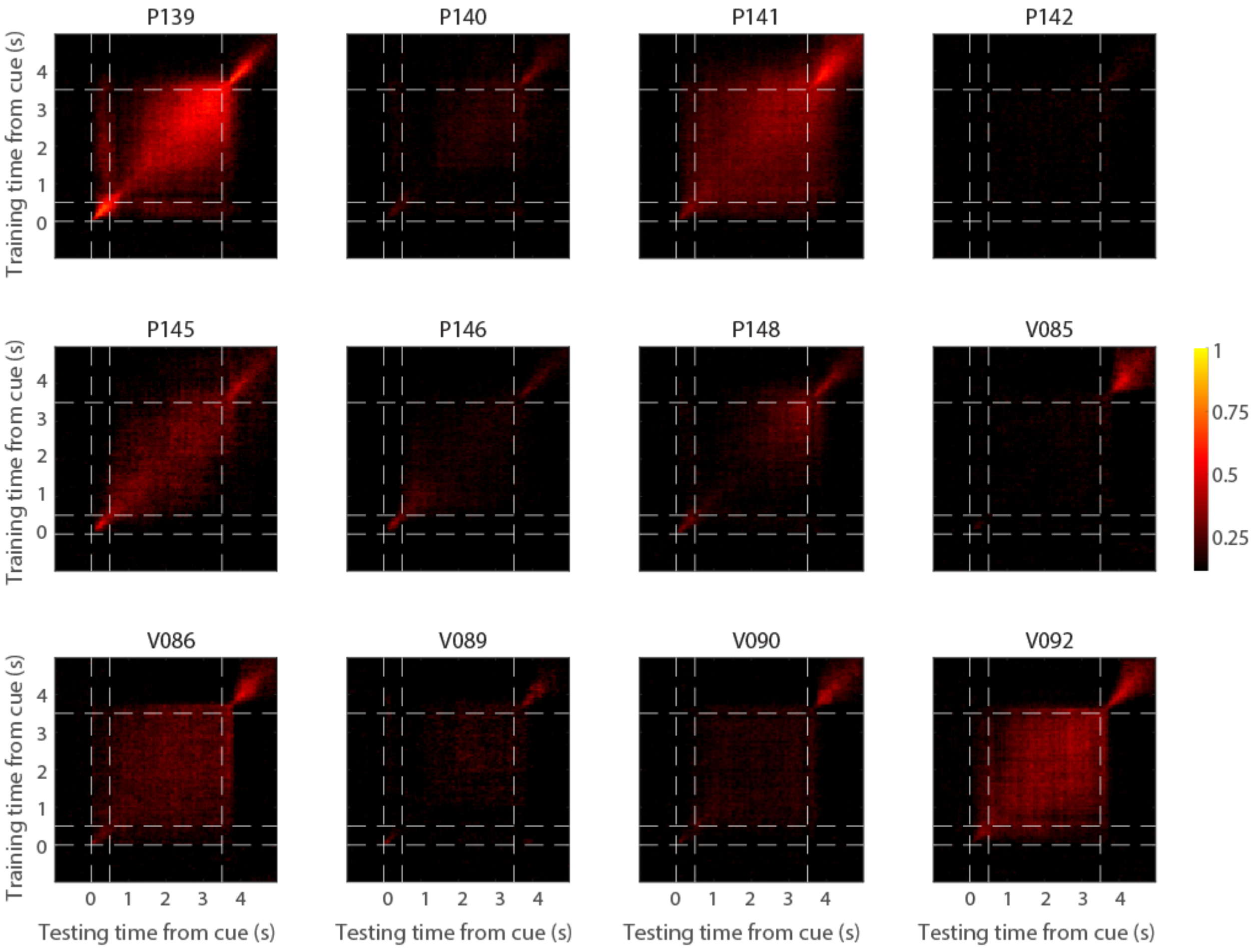

Figure S4. Cross-temporal decoding, related to Figure 2. Mean cross-temporal decoding for all recording sessions. The conventions are the same as in Figure 2 (central panel).

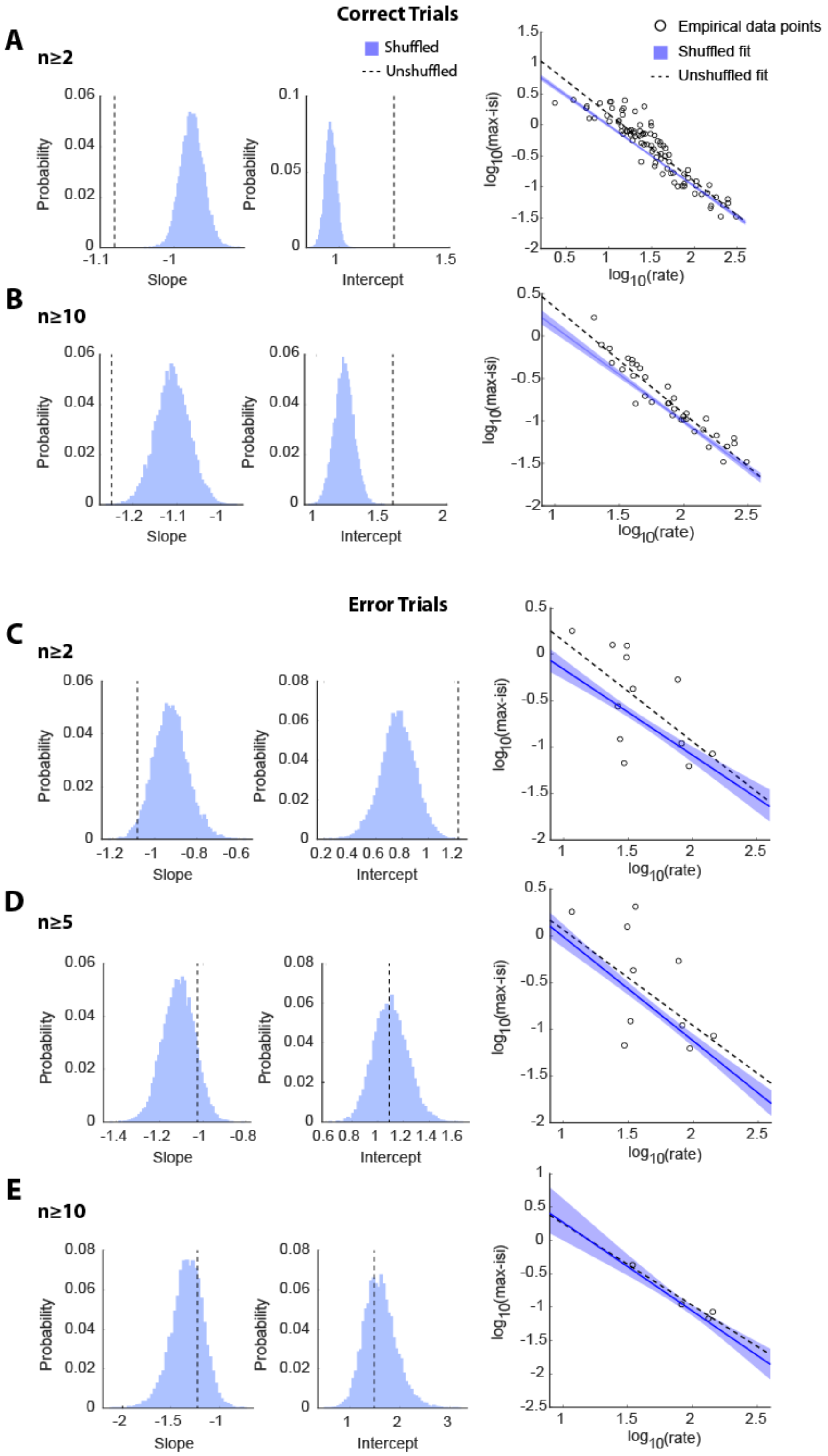

Figure S5. ISI analysis in populations of varied size and in error trials, related to Figure 3. (A-B) Same as in Fig. 3I-K, for ISIs computed from populations of at least 2 (A) or 10 neurons (B). (C-E). Same as above, but for error trials of populations with at least 2 (C), 5 (D), and 10 (E) neurons.

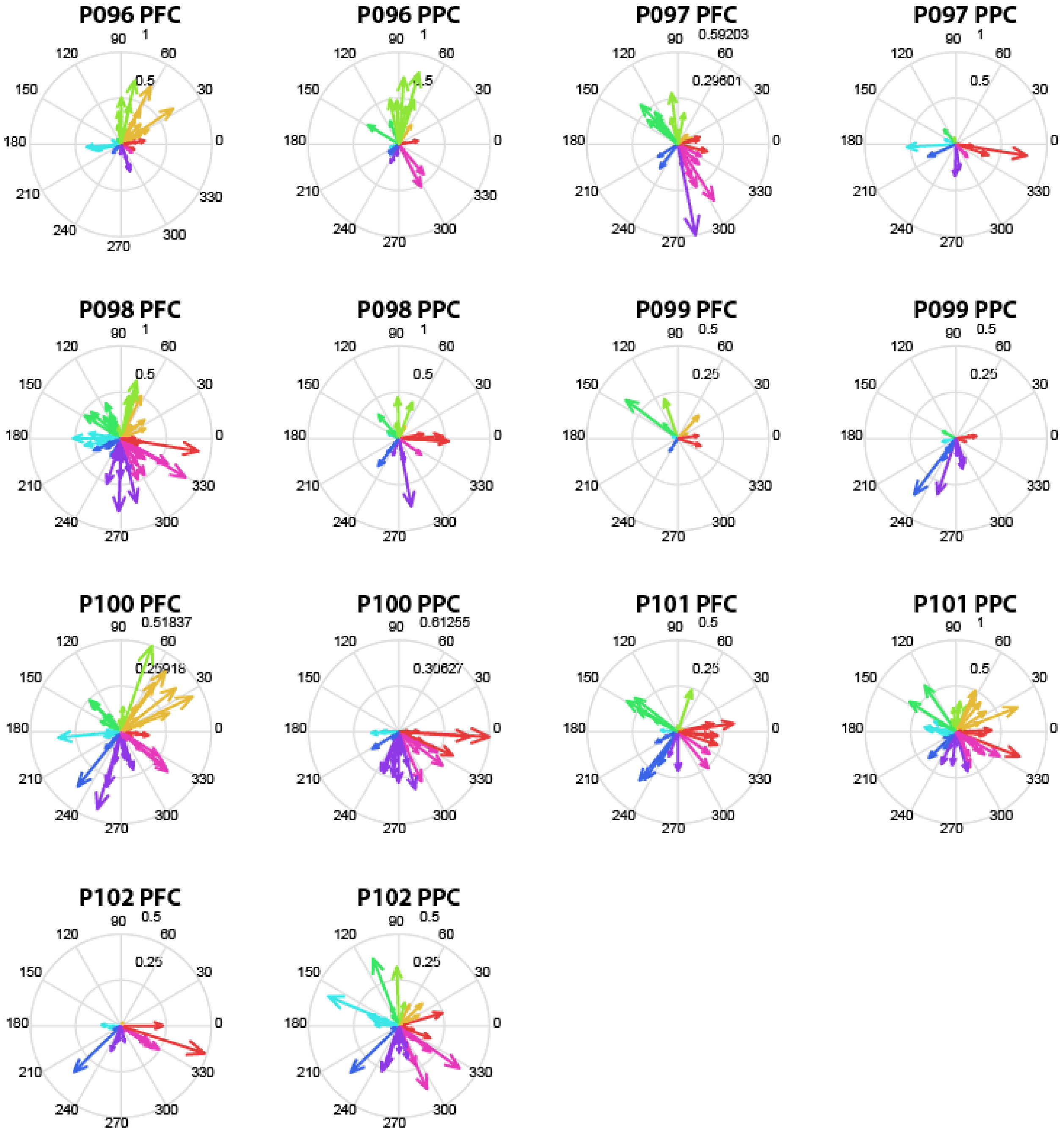

Figure S6. Compass plots for simultaneous PFC-PPC recordings, related to Figure 4. Compass plots are shown for all neurons recorded simultaneously in sessions with PFC-PPC recordings.

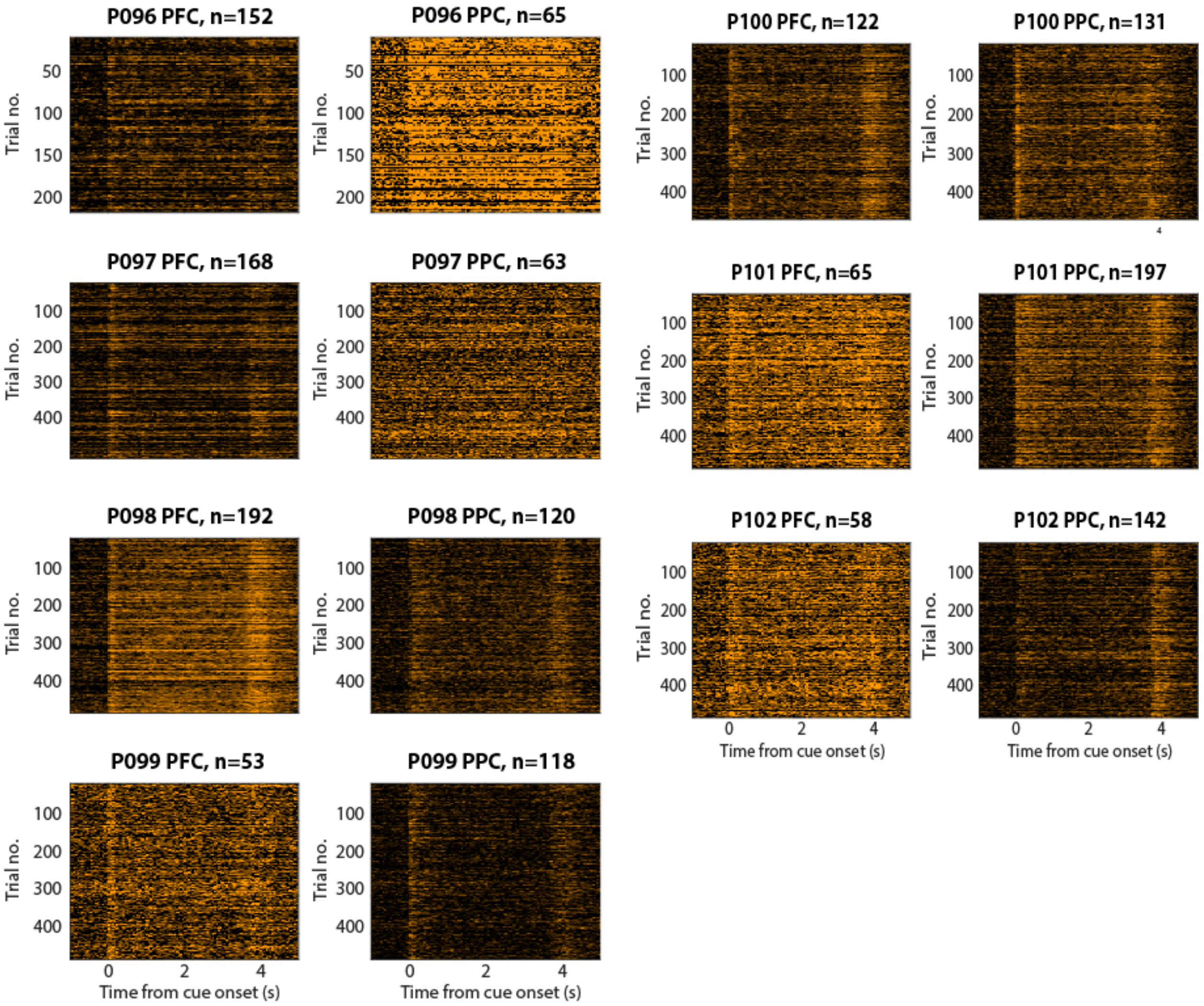

Figure S7. Single-trial classifier confidence for simultaneous PFC-PPC recordings, related to Figure 4. Single-trial classifier confidence is shown. Color scale as in Fig. 4A.

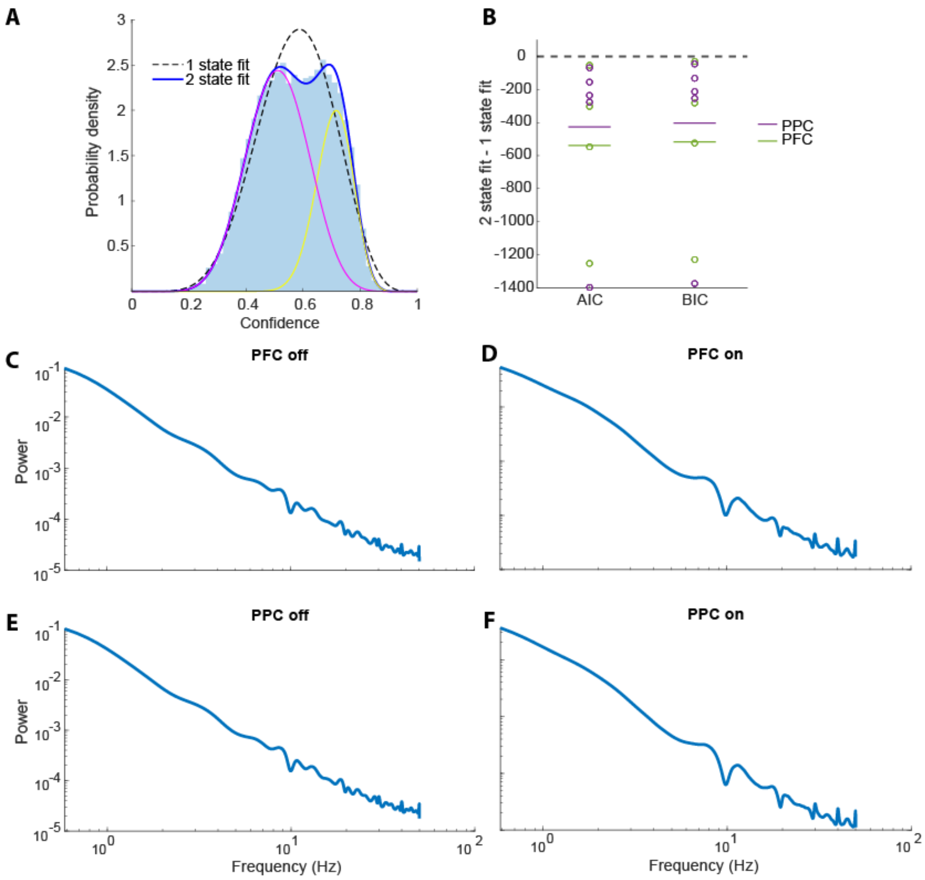

Figure S8. Characteristics of on- and off-states, related to Figure 4. (A). Example histogram of confidence values for one cue location from an example PFC session. The dashed line shows the 1-state beta fit while the solid blue lines demonstrate a 2-state fit. The magenta and yellow lines show the components for the two-state model. (B). Akaike Information criterion (AIC) and Bayesian Information Criterion (BIC) values for 1- and 2-state fits for all PPC and PFC sessions. (C-F). Welch power spectral density is plotted for on- and off-states in the prefrontal and posterior parietal cortex.

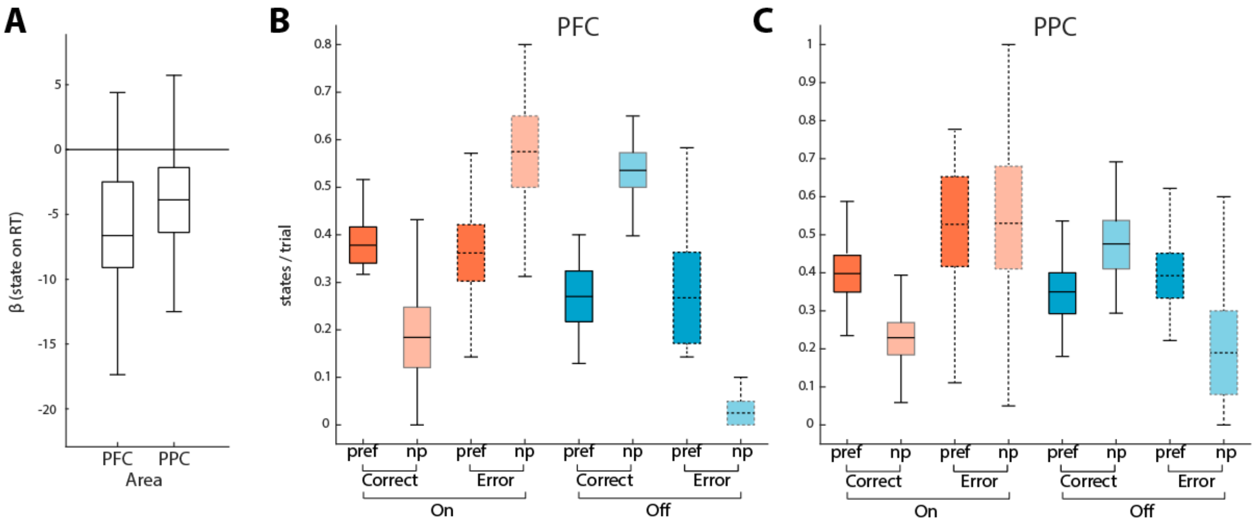

Figure S9. Relationship of on- and off-states with behavior, related to Figure 4. (A). Regression coefficient values for on- and off-states with relation to reaction time (RT). Negative values indicate shorter reaction times during on-states. (B). Number of on- (red) and off-states (blue) in the PFC per trial, for correct (solid boxes) and error trials (dotted boxes), when the stimulus appeared at the overall preferred location of the site (pref - darker colors) or non-preferred location (np - lighter colors). (C). Same as B for the PPC.

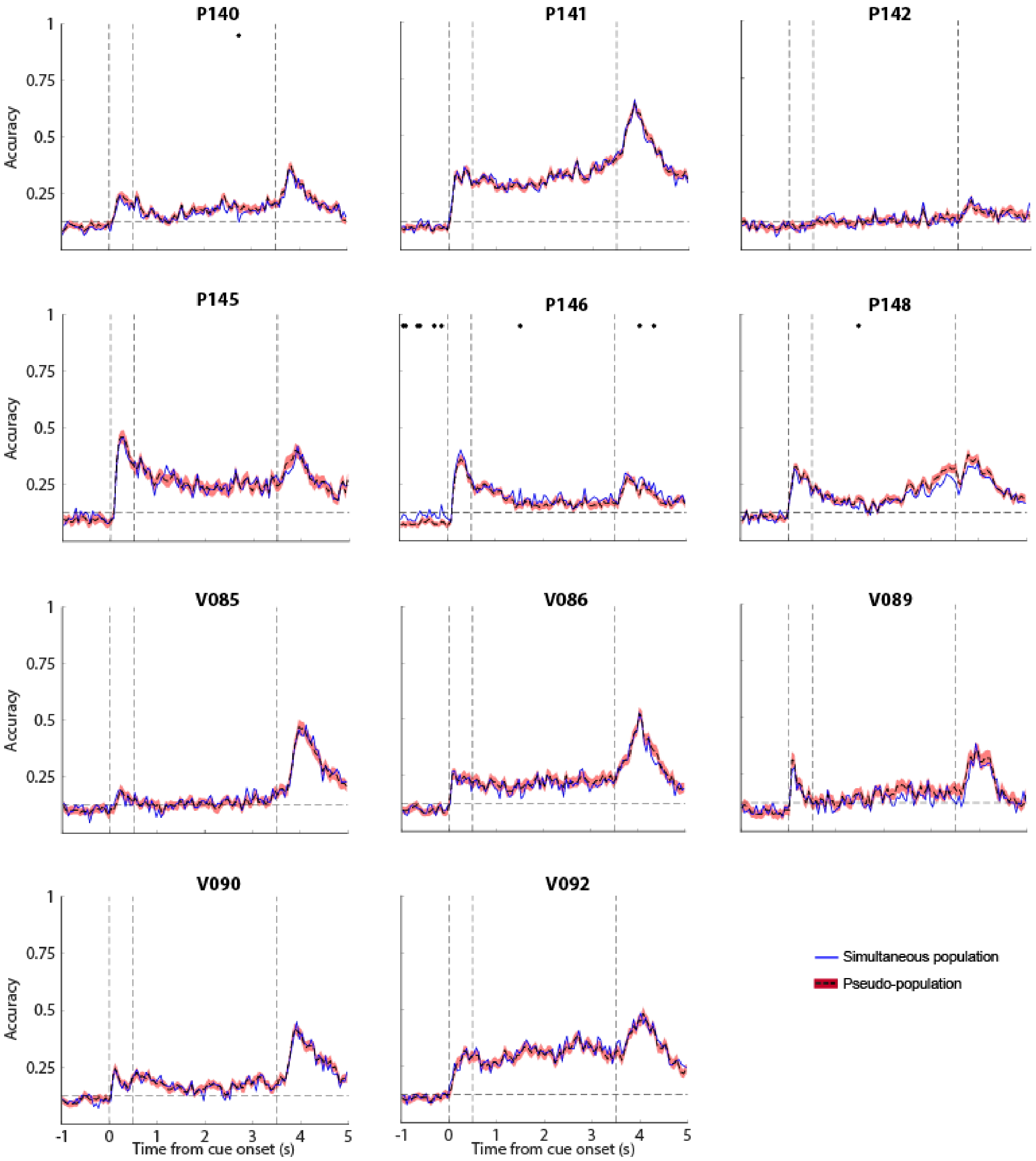

Figure S10. Decoding of simultaneous and pseudo-populations, related to Figure 5. Mean decoding performance is plotted for PFC recording sessions for populations of neurons obtained simultaneously vs. pseudo-populations constructed by splicing different trials together. Conventions are the same as in Fig. 5.
